# Smartwatch and physical education exam stress: the role of exercise self-efficacy and physical self-concept

**DOI:** 10.3389/fpsyt.2026.1882938

**Published:** 2026-07-22

**Authors:** Qi Liu, Hongli Yang, Qi Cui, Jihe Chen, Bing Shi

**Affiliations:** 1 School of Teacher Development, Shaanxi Normal University, Xi’an, China; 2 College of Education for the Future, Beijing Normal University, Zhuhai, China; 3 School of Education, Shanghai Jiao Tong University, Shanghai, China; 4 The Faculty of Education, Southwest University, Chongqing, China; 5 Physical Education School, Shaanxi Normal University, Xi’an, China

**Keywords:** adolescent health, artificial intelligence, exercise self-efficacy, physical self-concept, smartwatch

## Abstract

Against the backdrop of increasing educational digitalization and growing concerns over adolescent physical health, and based on social cognitive theory, this study examines the associations between attitudes toward smartwatch usage and the stress of junior high school students in the High School Entrance Examination Physical Education (PE examination), and further explores its dynamics through the correlational roles of exercise self-efficacy and physical self-concept. Structural Equation Modeling (SEM) analysis reveals that attitudes toward smartwatch use are directly negatively associated with PE examination stress (β = -0.197) and are also indirectly associated with lower stress through their positive associations with exercise self-efficacy (indirect effect = -0.108) and physical self-concept (indirect effect = -0.172). In addition, decision tree analysis using the CHAID algorithm identifies physical self-concept as the most critical associated predictive variable. Exercise self-efficacy and attitude toward smartwatch use serve as subsequent hierarchical factors. The findings suggest that smartwatch usage is negatively associated with examination stress by providing objective data feedback and functioning as a “digital mirror”, which is associated with boosting students’ confidence in exercise and body awareness. The results offer empirical evidence for the application of smart technology in school physical education and propose a “data-driven” correlational framework for understanding adolescents’ physical examination stress. Future research could incorporate longitudinal designs and cross-cultural comparisons to further validate the educational relevance of smartwatches, and should also examine potential negative effects such as data-driven anxiety or social comparison.

## 1 Introduction

The Physical Education Examination for High School Selection (PE examination) was originally instituted by the Chinese government (1). It was designed as an institutional strategy to enhance adolescent health, with the aim of compelling schools, families, and students to prioritize daily physical activity (2). China has gradually raised the scores of physical fitness tests to a level comparable to those of Chinese, mathematics, and English in the high school entrance examination, aiming to improve school physical education (3). However, China’s educational context is highly competitive, the stress associated with physical examinations among junior high school students is a real and increasingly severe phenomenon (4). It is also influenced by Confucian values, which emphasize academic achievement as a primary path for social mobility (5). As a result, this well-intentioned policy has inadvertently become a notable source of stress for junior high school students. This appears to contradict the policy’s original purpose of fostering physical and mental well-being.

China’s “Double Reduction” policy has significantly reduced academic workload and after-school tutoring demands (6, 7). While primarily aimed at alleviating academic stress, this policy has inadvertently increased attention to non-academic areas, including physical education. With less time spent on traditional academic subjects and tutoring, students and parents are now more focused on the PE examination as a key component of high school admission. This policy shift creates both an opportunity and a need for technology-based solutions—such as smartwatches—that can help students monitor and improve their physical fitness efficiently without adding excessive time burden. Thus, examining the role of smartwatch attitudes in mitigating PE examination stress is particularly relevant in the current policy context.

The emergence of smartwatches offers a novel technological pathway that may help address this contradiction by directly addressing the psychological roots of examination stress. Traditional physical education relies heavily on professional teacher guidance based on subjective observation and experience. Modern smartwatches integrate multimodal biosensor systems that can precisely track key physiological metrics, including heart rate variability, exercise intensity, and calorie expenditure (8). More importantly, these devices use machine learning-based data analysis to process biometric data and generate personalized, real-time feedback, such as intensity adjustment recommendations and virtual achievement rewards (9). This creates a “digital mirror” that provides students with objective, visualized performance assessments. Importantly, smartwatch-generated data and teacher guidance are not mutually exclusive but rather complementary. Teachers can use students’ objective metrics to refine their instructional decisions, offer more targeted support, and enhance the human elements of encouragement and pedagogical adaptation. Together, this integrated approach is potentially associated with reduced uncertainty and anxiety linked to subjective evaluation alone, thereby transforming stressful exam preparation into a manageable, data-informed process without replacing the irreplaceable role of professional teaching.

Importantly, the interpretation of smartwatch-generated bodily data is not purely objective or uniform across individuals. From an interoceptive perspective, the sense of the physiological condition of the body—including heart rate, breathing, and exertion—is inherently subjective and shaped by prior emotional and cognitive states (10, 11). Smartwatches provide external, quantified representations of these internal signals, creating a feedback loop between bodily sensation and digital reflection. However, this loop is bidirectional and context-dependent: an adolescent’s existing psychological state (e.g., anxiety, body dissatisfaction, or self-esteem) can color how they interpret their heart rate or step count, and conversely, seeing those metrics can in turn shape their emotional and bodily experience (12). Thus, smartwatch feedback should not be viewed as simple “objective information” that uniformly reduces uncertainty. Instead, it operates within a broader mind-body framework, where digital data are filtered through and interact with each student’s psychological and relational context.

These devices have introduced novel technological pathways that may be associated with reduced physical examination stress among junior high school students. Smartwatches have undergone a revolutionary transformation from fashion accessories to professional health monitoring devices. These wearable devices integrate multimodal biosensor systems to achieve precise capture and continuous monitoring of key physiological indicators (8). They can accurately track students’ heart rate variability, gait characteristics, exercise intensity, calorie expenditure, and other critical physiological metrics (9).

More importantly, machine learning-based data analysis systems in the smartwatch can foster individuals’ exercise self-efficacy. This refers to their confidence in successfully performing specific exercise tasks. These systems can also enhance physical self-concept, which is the overall perception and evaluation of one’s own physical abilities and appearance. Specifically, by providing real-time, personalized feedback and virtual rewards, the smartwatch helps students experience repeated success in small exercise goals, thereby strengthening their exercise self-efficacy. At the same time, accumulating these positive exercise experiences further contributes to a more positive physical self-concept (13). Both psychological factors are potentially associated with lower levels of PE examination stress. The system can process biometric data to generate personalized exercise feedback reports, including recommendations for intensity adjustment, periodic progress notifications, and motivational content such as virtual achievement rewards (14). This real-time, objective, and visual data feedback mechanism not only overcomes the limitations of subjective experience-based guidance in traditional physical education but also provides adolescents with quantifiable performance assessments. However, there remains a lack of clear empirical research on whether and how smartwatch use is associated with junior high school students’ physical examination stress through psychological mechanisms such as exercise self-efficacy and physical self-concept.

To date, the rapid popularization of smartwatches among adolescents provides practical opportunities for their adoption to benefit adolescents’ exercise and be associated with lower stress in the PE examination. The findings could extend the application of smartwatches technology in educational psychology while offering practical guidance for schools and families to optimize adolescents’ physical education experiences through technological means. Based on these considerations, this study aims to integrate social cognitive theory to examine the impact of smartwatch usage on junior high school students’ beliefs about physical examinations, with a specific focus on analyzing the chain-mediating roles of exercise self-efficacy and physical self-concept in this process. Specifically, the study seeks to address the following research questions,

Q1: Can smartwatch usage significantly alleviate physical examination stress among junior high school students?
Q2: Do exercise self-efficacy and physical self-concept play chain mediating roles in this influence process?

By addressing these questions, the research will contribute to a deeper understanding of how technology can be leveraged to support students’ psychological well-being and performance in physical education contexts. The results may inform the development of targeted interventions that harness smartwatches to foster healthier attitudes toward physical examinations among adolescents.

## 2 Literature review and research hypothesis

### 2.1 The PE examination stress

Since 2007, China has gradually implemented the Physical Education Examination for High School Selection (PE examination) as a key component of the junior high school evaluation system (15). In response to the declining physical health of adolescents, many regions have raised the weight of PE examination scores to 50–100 points, placing it on par with core subjects like Chinese and mathematics (16). While this reform promotes physical activity, it has also imposed unprecedented exam-related stress on students. The highly competitive, exam-oriented learning environment (17), combined with rising societal concerns over myopia, obesity, and declining fitness (18), has further intensified this pressure. Consequently, many junior high school students experience significant psychological stress during PE examinations, manifesting as anxiety, reduced performance, and even long-term aversive attitudes toward physical activity. Addressing this stress has thus become an urgent practical issue requiring effective, evidence-based interventions.

### 2.2 AI technology and physical exercise under social cognitive theory

Social Cognitive Theory (SCT) posits that human behavior is shaped by the dynamic and reciprocal interaction among three key factors: personal cognition, behavior, and environment. According to this theory, changes in any one factor can influence the other two, and these interactions continuously affect individual functioning and behavioral outcomes. In this study, these three components are operationalized as follows: smartwatch usage attitude represents the environmental factor, exercise self-efficacy and physical self-concept represent personal cognitive factors, and PE examination stress (PEES) represents the behavioral outcome.

As part of the environment, intelligent smartwatches can monitor users’ physiological data (e.g., heart rate, movement accuracy) in real time, providing immediate feedback as observable behavioral outcomes to facilitate self-regulation (19). They also offer algorithm-driven personalized training plans, ensuring that exercise routines align with individual needs and environmental conditions (20). Thus, smartwatches (environment) show associations with students’ cognitive beliefs (personal), which in turn are associated with their stress responses (behavior). Through this mechanism, the application of AI technology in physical exercise enhances the scientific rigor and sustainability of workouts.

Such technology not only helps adolescents better understand their own emotions and develop self-regulation skills but also effectively reduces the risk of psychological issues such as anxiety and stress associated with physical exercise (21). Therefore, this study hypothesizes that,

H1: Attitude toward smartwatch usage is negatively associated with PE examination stress.

### 2.3 Exercise self-efficacy

Exercise self-efficacy refers to an individual’s confidence in persisting with exercise and overcoming difficulties, which not only partly determines their motivation to participate in physical activity but also serves as one of the strongest predictors of exercise behavior (22). According to Social Cognitive Theory (SCT), human behavior is shaped by the dynamic interaction among personal cognition, behavior, and environment (23). In this study, these three components are operationalized as follows: smartwatch usage attitude represents the environmental factor; exercise self-efficacy and physical self-concept represent personal cognitive factors; and PE examination stress represents the behavioral outcome.

Smartwatches, as environmental tools, provide real-time physiological feedback and personalized training plans, thereby influencing students’ cognitive beliefs, which in turn shape their stress responses. Importantly, smartwatch feedback and teacher guidance are not oppositional forces but complementary elements within the educational environment. While smartwatches offer objective, continuous data that individualize feedback, teachers provide contextualized interpretation, emotional support, and pedagogical scaffolding that technology alone cannot deliver.

The optimal educational setting likely involves a synergistic relationship: teachers leverage smartwatch data to inform their instructional decisions, and students use the same data to self-regulate under teacher supervision. Therefore, this study does not advocate for replacing human instruction with technology, but rather for understanding how smartwatches can be integrated into existing teacher-led physical education to enhance its psychological and behavioral benefits. Through this mechanism, the application of AI technology in physical exercise enhances the scientific rigor and sustainability of workouts.

Adolescents in the rising stage of puberty, middle school students are at a critical developmental period. This phase plays a foundational role in shaping lifelong health behaviors and mental well-being, while also being a high-risk period for psychological development, making them more susceptible to physical health issues (24). Smartwatches, through their unique functional design, provide multifaceted support for fostering exercise self-efficacy in adolescents. Among these features, quantified monitoring transforms abstract exercise concepts into concrete numerical metrics, allowing users to visualize their progress. This tangible sense of achievement directly reinforces the belief of “I can do it” (13). Instant feedback upon goal achievement, such as vibration alerts and virtual rewards, triggers the brain’s pleasure response and helps establish a conditioned reflex between exercise and positive emotions (25). Therefore, this study hypothesizes,

H2: Attitude toward smartwatch usage is positively associated with middle school students’ exercise self-efficacy.

A 2025 experiment published in JMIR mHealth demonstrated that middle school students using smartwatches showed significant improvement in exercise self-efficacy (measured by the ESE scale) after eight weeks, particularly in the dimensions of “overcoming time constraints” and “resisting fatigue” (26). Fitbit data also revealed that 78% of long-term wearers were more confident in their ability to maintain exercise, while 68.3% of current users and 70.2% of former users reported initial increases in their activity levels (27). Additionally, the data visualization feature of smartwatches reduces uncertainty about exercise and diminishes psychological barriers (28). These enhancements in exercise self-efficacy are expected to be associated with lower PE examination stress, because students who feel more capable of persisting through training and overcoming physical challenges are less likely to perceive the exam as threatening or beyond their control. Therefore, this study hypothesizes,

H3: Exercise self-efficacy mediates the association between smartwatch usage attitude and PE examination stress.

### 2.4 Physical self-concept

Physical self-concept refers to an individual’s comprehensive perception and evaluation of their physical characteristics, bodily functions, and athletic capabilities, encompassing subjective assessments of body shape, physical fitness, and health status (29). A 14-day longitudinal study revealed that when data reflects improvements in physical fitness or the achievement of exercise goals, it reinforces a sense of “bodily control” and fosters greater self-identification with one’s athletic potential (30). Research by Kamal Basha et al. (31) demonstrated that wearable devices such as smartwatches can significantly influence users’ cognition and experience of their physical state (31).

By providing real-time monitoring and feedback on physiological data, these devices help users develop a clearer body awareness and enhance their ability to perceive their own health status. Additionally, “understanding oneself through data” strengthens users’ sense of control over their physical functions, enabling them to better comprehend their bodies and purposefully design plans to improve overall health (32). Furthermore, smartwatches show potential in assisting with the management of specific physical conditions, such as tracking menstrual cycles, monitoring chronic disease indicators, or optimizing athletic performance. These features encourage users to take a more proactive role in their health management, fostering a more positive body image and healthier lifestyle (33). Therefore, this study hypothesizes,

H4: Attitude toward smartwatch usage is positively associated with middle school students’ physical self-concept.

Smartwatches provide middle school students with visualized feedback on their physical state by continuously tracking exercise data and physiological metrics (34). According to Social Cognitive Theory (SCT), environmental factors—such as smartwatch feedback—shape personal cognitive beliefs, which in turn influence behavioral outcomes like examination stress. Within this framework, physical self-concept is conceptualized as a global, affect-laden personal cognition that develops from repeated mastery experiences. When students observe gradual improvements in their exercise performance via smartwatches, their task-specific confidence (exercise self-efficacy) is enhanced. Over time, this specific confidence generalizes into a more positive overall evaluation of their body and athletic competence, namely physical self-concept.

This positive physical self-concept then serves as a psychological buffer against PE examination stress. Students who hold a more favorable physical self-concept are less likely to engage in negative social comparison with peers, less prone to worry about body image or physical competence during exams, and more inclined to interpret physical challenges as manageable rather than threatening. Additionally, real-time stress assessment through heart rate variability and electrodermal activity technologies enables smartwatches to detect anomalies and automatically suggest interventions such as breathing exercises (35).

Thus, consistent with SCT’s principle of hierarchical self-belief development, smartwatch attitude is not directly associated with lower stress solely through raw data. Instead, it follows a sequential chain: first enhancing exercise self-efficacy (task-specific confidence), then strengthening physical self-concept (global self-evaluation), and finally reducing PE examination stress. Therefore, this study hypothesizes:

H5: Physical self-concept mediates the association between smartwatch usage attitude and PE examination stress.

**Figure 1:**
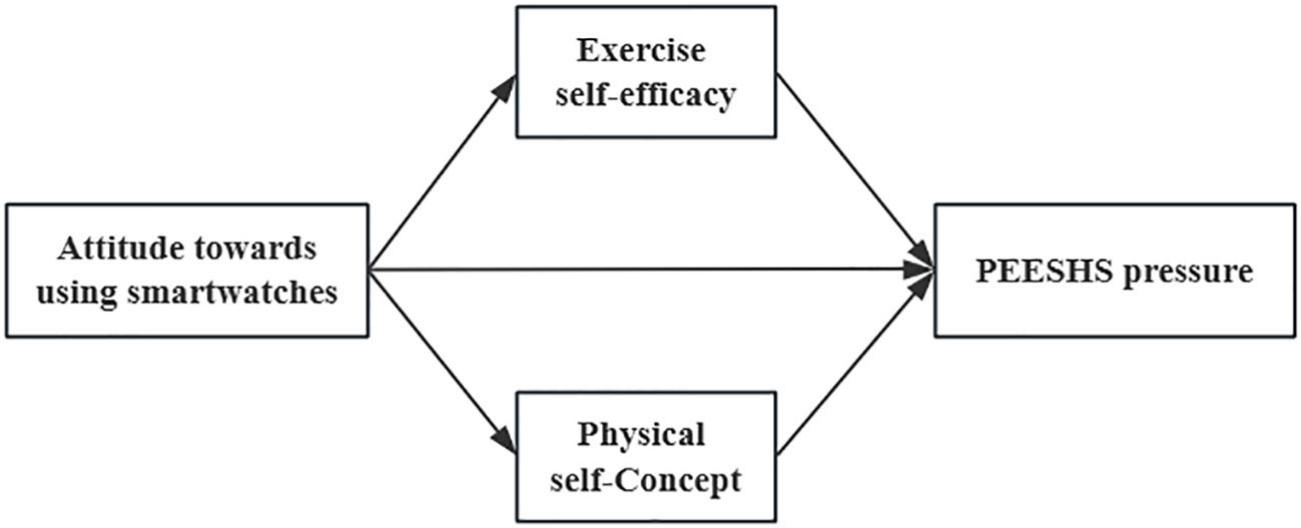
Research model based on social cognitive theory.

Based on Social Cognitive Theory, this study constructs a theoretical model. The model operationalizes three core components. Attitude toward smartwatch usage represents the environmental factor. Exercise self-efficacy and physical self-concept represent personal cognitive factors. PE examination stress represents the behavioral outcome. Smartwatches provide real-time feedback such as heart rate, step count, and personalized guidance. This feedback helps students accurately assess their exercise capabilities. It reduces uncertainty and supports mastery experiences. These effects strengthen exercise self-efficacy, which is task specific confidence. Enhanced self-efficacy then generalizes into a more positive physical self-concept, which is a global self evaluation. A positive physical self-concept ultimately lowers PE examination stress by reducing threat appraisal and fear of negative evaluation. The model therefore proposes that smartwatches are associated with lower stress through a sequential chain from environment to specific cognition to global cognition to behavioral outcome (**Figure 1**).

## 3 Methods

### 3.1 Data collection

China’s PE examination has been implemented in most provinces and carries significant weight in high school admissions. Preliminary research revealed that many students experience fear, anxiety, and stress related to the PE examination, particularly in Guangdong. Such stress may not only hinder their exam performance but also negatively impact their long-term attitudes toward physical exercise. Additionally, Guangdong’s relatively high economic development means many students can afford smartwatches.

Therefore, this study conducted paper-based surveys across three junior high schools in Guangdong Province. The three schools were selected using convenience sampling, all of which had officially implemented the PE examination policy and permitted students to use smartwatches during daily training. A cluster sampling method was adopted: all ninth-grade classes from the three schools were included, resulting in a total of 15 classes (5 classes per school) with 600 students. During questionnaire design, we strictly adhered to Chinese-English bilingual translation protocols, using clear and precise wording to ensure respondents accurately understood each question, thereby minimizing subjective bias due to misinterpretation.

The inclusion criteria were: (a) being a ninth-grade student, (b) owning a smartwatch, and (c) providing voluntary participation with parental consent. The exclusion criteria were: (a) absence on the survey day, (b) incomplete questionnaire, or (c) withdrawal of consent. Questionnaires were distributed and collected on-site by trained research assistants (not classroom teachers) during homeroom periods to ensure standardized administration and reduce social desirability bias. All questionnaires were completely anonymous, and the collected data were used solely for academic research purposes.

All participants completed written informed consent. Because this study included minors, written consent was also obtained from their parents or legal guardians. No missing data were present in the collected questionnaires, as all items were required to be answered on site and research assistants checked each questionnaire for completeness before collection. Invalid questionnaires (N = 73) were identified based on two criteria: (a) patterned responding (e.g., selecting the same option for all items, i.e., straight-lining), or (b) logically inconsistent answers (e.g., contradictory responses to reverse-coded items). This study received ethical approval from the Academic Committee of Shaanxi Normal University (Ethics Approval Number: 202616036).

### 3.2 Analysis procedure

This study utilized SPSS 26 and SmartPLS 4 for data analysis. SPSS 26 was employed for descriptive analysis and decision tree construction, while SmartPLS 4 was used to establish structural equation modeling (SEM) and test research hypotheses. Specifically, PLS-SEM is particularly suitable for predictive applications and does not require normally distributed data assumptions. Its flexibility in handling non-normal data distributions and complex model structures has led to its increasing adoption across various disciplines (36).

This study introduced a classification decision tree model based on the CHAID (Chi-squared Automatic Interaction Detector) algorithm from machine learning. The CHAID tree was grown using the following parameters: the significance level for both node splitting and category merging was set at α = 0.05, with Bonferroni adjustment for multiple comparisons to control Type I error. The maximum tree depth was limited to 3 to enhance interpretability. The minimum number of cases for parent nodes was set to 100, and for child nodes to 50, ensuring statistical stability of subgroups. No post-pruning rules were applied. No cross-validation or training/test split was used, as the primary purpose was to explore the hierarchical associative structure among variables rather than to optimize predictive accuracy for external generalization. The tree diagram clearly illustrates the interactions among factors while indicating whether a given factor holds significance within specific subgroups (37).

### 3.3 Measurement instrument

The survey questionnaire consisted of three sections. The first section contained instructions explaining the purpose of the survey and emphasizing confidentiality. The second section collected students’ demographic information, including gender, grade level, type of smartwatch worn, and cumulative duration of smartwatch usage. The third section incorporated established measurement scales to assess key constructs, including attitudes toward smartwatch usage, perceived PE examination stress, exercise self-efficacy, and physical self-concept. To ensure measurement consistency, all four scales employed a 5-point Likert scale (1 = strongly disagree, 5 = strongly agree), with higher scores indicating stronger perceived agreement with each measured item.

All scales underwent a standardized translation and back-translation procedure to ensure cultural appropriateness for Chinese junior high school students. A pilot study was conducted with 45 ninth-grade students (not included in the final sample) to assess clarity and comprehensibility, resulting in minor wording revisions to three items. All four scales are published and publicly available academic instruments. Detailed measurement items are presented in the **Appendix**. Proper citations to the original sources are provided in the references.

Attitude toward using smartwatches (ATUS) was adapted from the Artificial Intelligence Attitude Scale (38). The four items measure students’ positive perceptions and acceptance of smartwatch technology. The scale’s Cronbach’s α was 0.804.

Perceived PE examination stress (PEES) was adapted from the physiological anxiety response section of the Exam Stress Scale (39). The ten items measure students’ physiological and psychological stress responses when anticipating the PE examination. The scale’s Cronbach’s α was 0.895.

Exercise self-efficacy (ESE) was adapted from the Exercise Self-Efficacy Scale (40). The nine items measure students’ confidence in persisting with exercise when facing various barriers. The scale’s Cronbach’s α was 0.887.

Physical self-concept (PSC) was adapted from Fraguela-Vale et al. (41). The six items measure students’ perceptions and evaluations of their own physical abilities and body image. The scale’s Cronbach’s α was 0.865.

### 3.4 Participant information

To better assess students’ perceived stress regarding the PE examination, the study focused on ninth-grade students in their second semester. The official PE examination was scheduled to be held on April 8, 2026. Since smartwatches are not permitted during the actual examination, the baseline survey was conducted from March 23 to March 31, 2026, during which participants completed the questionnaire. Before distributing the questionnaires, researchers provided instructions to ensure students answered all questions carefully. A total of 600 questionnaires were collected. After excluding 73 invalid responses, 527 valid questionnaires remained, yielding an effective response rate of 87.83%.

**Table 1** presents the demographic characteristics of the sample, including gender, smartwatch brands, frequency of smartwatch use, and weekly exercise sessions tracked via smartwatches. Among the 527 participants, gender distribution was balanced, with 56.4% male and 43.6% female.

**Table 1:** Participant information (N = 527).

Variable	Category	N	%
Gender	Male	297	56.4
	Female	230	43.6
Smartwatch brands	Huawei	163	30.9
	Xiaomi	133	25.2
	Apple	104	19.7
	OPPO	65	12.3
	Other	62	11.8
Usage frequency	Every day	69	13.1
	Half a day	204	38.7
	Every class	140	26.6
	At any time	114	21.6
Exercise tracking	0-2	51	9.7
	3-5	133	25.2
	6-8	205	38.9
	≥9	138	26.2

## 4 Results

### 4.1 Common method bias

Since the data were collected through self-reported measures, we employed two complementary approaches to assess potential common method bias. First, Harman’s single-factor test was conducted. The results revealed five factors with eigenvalues greater than 1. The first factor accounted for 18.570% of the variance, which is below the critical threshold of 40% (42). Second, we examined the variance inflation factor (VIF) to detect potential multicollinearity, which can also indicate common method bias. All VIF values ranged from 1.324 to 2.878, below the recommended threshold of 3.3. Taken together, these results indicate that common method bias was within an acceptable range and would not significantly affect the statistical findings.

### 4.2 Descriptive statistics and normality test

Before assessing reliability and validity, we examined the descriptive statistics of the four key constructs to understand their distributions. **Table 2** presents the means, standard deviations (SD), skewness, and kurtosis for attitude toward using smartwatch (ATUS), PE examination stress (PEES), exercise self-efficacy (ESE), and physical self-concept (PSC). The skewness and kurtosis values for all constructs were within the acceptable range of -2 to +2, indicating that the data approximated a univariate normal distribution.

**Table 2:** Descriptive statistics of key constructs (N = 527).

Construct	Mean	SD	Skewness	Kurtosis
Attitude towards using smartwatch	3.205	0.942	-0.409	-0.034
PE examination stress	2.075	0.790	1.415	0.999
Exercise self-efficacy	3.764	0.779	0.234	-0.486
Physical self-concept	3.841	0.812	0.001	-0.541

### 4.3 Reliability and validity tests

The construct validity of a scale reflects the degree to which the measurement tool accurately assesses its theoretical construct, serving as a key indicator of the validity of the research method (43). Following the recommendations of Fornell & Larcker (44), the acceptable lower limit for factor loadings was set at 0.60, and the minimum standard for KMO was 0.65. Two items in PE examination stress and one item in the Exercise self-efficacy scale had factor loadings of less than 0.5, which were not included in the subsequent analysis (45, 46).

The results in **Table 3** showed that all item factor loadings ranged between 0.652 and 0.844, meeting the criteria. The KMO values for each factor ranged from 0.779 to 0.924, all exceeding 0.65, indicating good construct validity for the five factors (44). For reliability testing, this study evaluated composite reliability (C.R.) and factor loading. As shown in **Table 3**, the C.R. values for each factor ranged from 0.824 to 0.898, and the average variance extracted (AVE) ranged from 0.561 to 0.626, meeting the standards proposed by Bollen (47) and Ramadani et al. (48). These results demonstrate that the five factors exhibit good internal consistency and discriminant validity.

**Table 3:** Measurement results of reliability and validity (N = 527).

Construct	Item	Factor loading	KMO	C.R	AVE
Attitude towards using smartwatches	ATUS1	0.844	0.779	0.824	0.626
	ATUS2	0.714			
	ATUS3	0.810			
	ATUS4	0.791			
PE examination stress	PEES1	0.756	0.923	0.898	0.578
	PEE2	0.788			
	PEES3	0.797			
	PEES4	0.832			
	PEES5	0.773			
	PEES6	0.668			
	PEES7	0.736			
	PEES8	0.719			
Exercise self-efficacy	ESE1	0.793	0.924	0.864	0.561
	ESE2	0.615			
	ESE3	0.813			
	ESE4	0.677			
	ESE5	0.761			
	ESE6	0.799			
	ESE7	0.755			
	ESE8	0.757			
Physical self-concept	PSC1	0.77	0.856	0.892	0.601
	PSC2	0.652			
	PSC3	0.774			
	PSC4	0.798			
	PSC5	0.838			
	PSC6	0.805			

### 4.4 Discriminant validity

This study employed the HTMT ratio and the Fornell-Larcker criterion to assess the discriminant validity of the measurement model. As shown in **Table 4**, the HTMT values for all latent variables were significantly lower than the threshold of 0.90 recommended by Henseler et al. (49), indicating satisfactory discriminant validity between constructs. Specifically, the HTMT value between Attitude towards using smartwatch and PE examination stress was 0.544, between Exercise self-efficacy and PE examination stress was 0.613, and between Physical self-concept and PE examination stress was 0.706. All these values were well below the discriminant threshold, confirming significant distinctiveness among the measured constructs.

**Table 4:** HTMT results (N = 527).

Variables	ATUS	ESE	PEESHSS	PSC
ATUS				
ESE	0.460			
PEESHSS	0.544	0.613		
PSC	0.488	0.596	0.706	

ATUS, Attitude towards using smartwatch; PEES, PE examination stress; ESE, Exercise self-efficacy; PSC, Physical self-concept.

This study assessed discriminant validity by comparing the square roots of the average variance extracted (AVE) for each factor with the Pearson correlation coefficients between factors (44, 50, 51). As shown in **Table 5**, the diagonal values represent the square roots of AVE for each factor, reflecting internal association strength, while the off-diagonal values indicate inter-factor correlation coefficients.

**Table 5:** Fornell-Larcker criterion Results (N = 527).

Construct	PEES	ATUS	PSC	ESE
PEES	**0.760**			
ATUS	-0.477	**0.791**		
PSC	-0.625	0.426	**0.775**	
ESE	-0.554	0.415	0.525	**0.749**

ATUS, Attitude towards using smartwatch; PEES, PE examination stress; ESE, Exercise self-efficacy; PSC, Physical self-concept.

The off-diagonal bold values indicate the inter-factor correlation coefficients.

### 4.5 Structural model analysis

**Figure 2:**
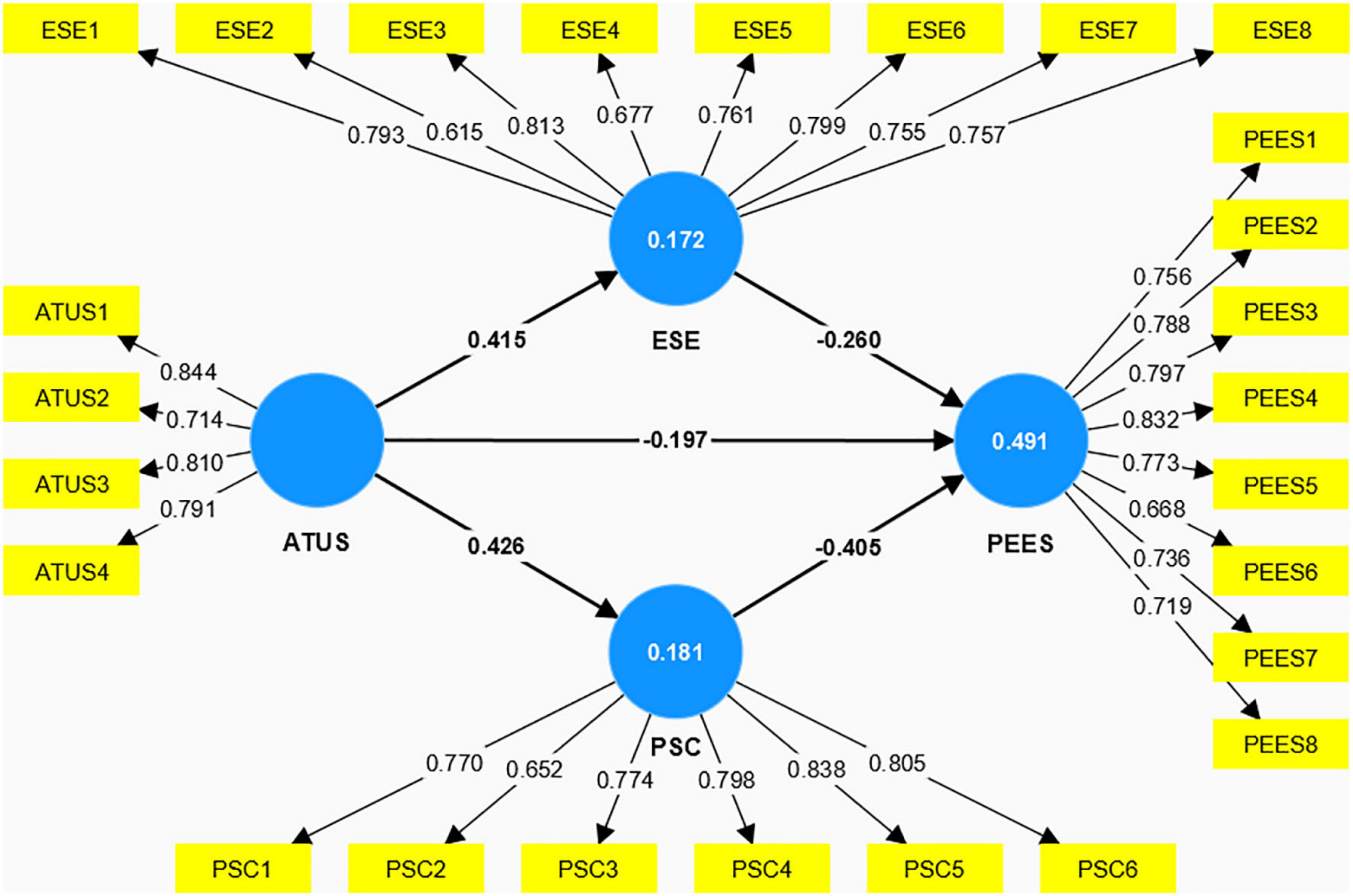
Standardized path coefficients of the PLS-SEM model. ATUS, Attitude towards using smartwatch; PEES, PE examination stress; ESE, Exercise self-efficacy; PSC, Physical self-concept.

After completing the reliability and validity tests of the measurement model, this study employed partial least squares structural equation modeling (PLS-SEM) to evaluate the structural model. Following the recommendations of Hair et al. (52) and Liu et al. (53), the study assessed model fit and predictive power using key indicators including the coefficient of determination (R²), adjusted R², effect size (f²), predictive relevance (Q²), and the standardized root mean square residual (SRMR). The analysis involved 5,000 bootstrap subsamples with two-tailed testing at a 5% significance level (95% bias-corrected confidence intervals), where hypothesis testing was evaluated using t-statistics and p-values. The results are presented in **Figure 2**.

Regarding explanatory power, the R² values for the three endogenous variables were 0.172 for exercise self-efficacy (ESE), 0.181 for physical self-concept (PSC), and 0.491 for PE examination stress (PEES). The adjusted R² values were 0.171, 0.180, and 0.488, respectively. According to Hair et al.’ (36)) criteria, the model demonstrated moderate explanatory power for PE examination stress. For effect sizes (f²), the contribution of attitude toward using smartwatch (ATUS) to ESE was 0.208 (medium), to PSC was 0.221 (medium), and to PEES was 0.059 (small); ESE contributed to PEES with an f² of 0.090 (small); PSC contributed to PEES with an f² of 0.217 (medium). Using the blindfolding procedure with an omission distance of 7, the Q² values were 0.098 for ESE, 0.095 for PEES, and 0.289 for PSC, all above zero, indicating the model’s predictive relevance. For model fit, the SRMR value of 0.070 was below the threshold of 0.08, suggesting acceptable model fit. All path coefficients and outer loadings reached statistical significance (p < 0.05).

**Table 6** summarizes the coefficients, T-values, and P-values of five path relationships. According to the significance criteria (T > 1.96, p < 0.05) proposed by Chen et al. (45), all five paths were statistically significant. The table clearly presents both the magnitude of each independent variable’s effect on dependent variables and the mediation effects of two indirect pathways.

**Table 6:** Inspection Results of the path (N = 527).

H	Path	β	T statistics	Upper CI	Lower %CI	P
H1	ATUS → PEES	-0.197	5.602	-0.267	-0.129	0.000
H2	ATUS → ESE	0.415	10.034	0.326	0.491	0.000
H3	ATUS → ESE → PEES	-0.108	5.055	-0.151	-0.067	0.000
H4	ATUS → PSC	0.426	11.484	0.350	0.495	0.000
H5	ATUS → PSC →PEES	-0.172	8.020	-0.217	-0.133	0.000

ATUS, Attitude towards using smartwatch; PEES, PE examination stress; ESE, Exercise self-efficacy; PSC, Physical self-concept.

### 4.6 Decision tree analysis of the CHAID algorithm

**Figure 3:**
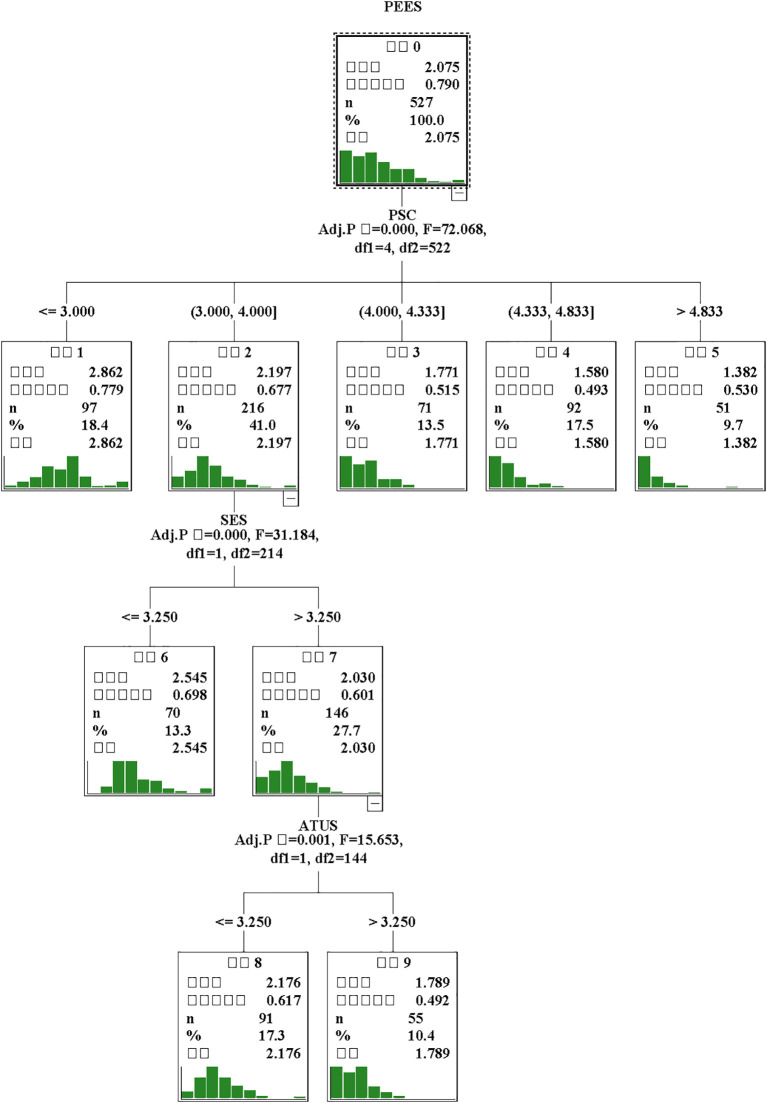
CHAID decision tree for predicting PE examination stress. ATUS, Attitude towards using smartwatch; PEES, PE examination stress; ESE, Exercise self-efficacy; PSC, Physical self-concept.

The classification decision tree model was constructed based on the CHAID parameters specified in section 3.2. Specifically, the minimum number of cases for parent nodes was set at 100, and for child nodes at 50 (not 400/200 as previously stated; this earlier description was a typographical error and has been corrected to align with the actual analysis settings). The resulting model consists of 3 layers and 10 nodes (including the root node), with 7 terminal nodes (**Figure 3**). The overall risk estimate of the model was 0.368 (SE = 0.034), representing the resubstitution error (i.e., the proportion of variance unexplained in the dependent variable, PEES). This value indicates that approximately 63.2% of the variance is correctly accounted for by the model within the current sample. However, because no cross-validation or independent test set was used, this risk estimate may be optimistic and should be interpreted with caution when generalizing to other populations. The model diagram demonstrates that each node’s classification exhibits good statistical significance (Bonferroni-adjusted p < 0.001).

The root node (first layer) of the decision tree is Physical self-concept, indicating that this variable shows the strongest statistical association with PE examination stress. The second layer further divides the highest category of Physical self-concept into two groups based on Exercise Self-Efficacy. The third layer reveals that Attitude towards using smartwatch is the next partitioning variable for Exercise Self-Efficacy. This hierarchical structure demonstrates the successive associative relationships among the variables and should not be interpreted as causal ordering. Among all categories, the range of (3, 4] for Physical self-concept has the highest proportion at 41.0%, significantly exceeding the other four categories. The second layer further divides the highest category of Physical self-concept into two groups based on Exercise Self-Efficacy, one with values ≤3.25 (accounting for 13.3%) and another with values >3.25 (27.7%). The third layer reveals that Attitude towards using smartwatch is the most direct influencing factor for Exercise Self-Efficacy. This variable is similarly categorized into two groups: ≤3.25 and >3.25. This hierarchical structure effectively demonstrates the progressive relationships among the variables in predicting PE examination stress.

## 5 Discussion

### 5.1 Manifestations of direct effect mechanisms

The attitude toward smartwatch usage demonstrates a significant direct negative association with PE examination stress. According to existing research, this association may be explained by the fact that when students wear smartwatches during training, real-time heart rate monitoring (e.g., HRV metrics on Apple Watch) and motion analysis functions provide immediate feedback on training performance. This “digital companionship” is associated with reduced students’ anxiety about uncertain exam outcomes (54). The functionalities of smartwatches are associated with students’ better ability to meet task requirements and improve their physical training performance (55).

At a deeper level, the significant association between smartwatch usage attitude and exercise self-efficacy suggests a psychological mechanism of “competence assurance.” The tracking data generated by smartwatches contribute to the formation of a techno-body, digitizing physicality and reshaping personal bodily cognition (56). This digitalized cognition is not only associated with user’s enhanced self-assessment of their exercise capabilities but also shows associations with the mental representation of physical activity through real-time feedback mechanisms. When users observe objective metrics such as step count, heart rate, and calorie consumption recorded by smartwatches, these data points are internalized as a form of competence assurance, showing lasting associations with subsequent exercise decisions and persistence (57). This makes smartwatches particularly relevant for middle school students in physical activities (58).

The significant association between smartwatch usage attitude and physical self-concept underscores the unique importance of body image among adolescents. The virtual representations conveyed through data are associated with enhanced users’ enjoyment of smartwatch interactions, thereby showing associations with their self-perception of physical capabilities (59). This finding suggests that smartwatches are not merely exercise-tracking tools but also play a role in being associated with adolescents’ bodily cognition. When these devices present user activity data through visualized dashboards, 3D models, or social sharing features, they may function as a “digital mirror” that potentially supports users’ ability to observe and evaluate their physical performance from a third-person perspective (60). This externalized self-representation is associated with reinforced body awareness and a more structured self-assessment process, particularly during critical developmental stages. It should be noted that this metaphor describes a theoretical possibility rather than a directly measured mechanism, as the present study did not assess how students actually view or interpret the visualized data.

### 5.2 The underlying logic of mediation effects

A positive attitude toward smartwatches is indirectly associated with lower perceived PE examination stress through its positive associations with exercise self-efficacy and physical self-concept. These devices track physical and physiological metrics to assess performance and recovery during exercise, which may provide more objective measurements compared to subjective self-reports (61). This data-driven feedback is associated with users’ clearer understanding of their exercise progress and stress fluctuations.

When smartwatches deliver real-time performance analytics, they show positive associations with users’ exercise self-efficacy—the confidence in achieving fitness goals—which in turn shows positive associations with increased physical activity (9). Moreover, prolonged smartwatch usage is associated with more consistent exercise habits (13), leading to improvements in body composition or physical fitness. These physiological changes show positive associations with physical self-concept, the cognitive and affective evaluation of one’s bodily capabilities and appearance.

The enhancement of these two intrinsic psychological factors is associated with a shift in individuals’ health behavior evaluation from social comparison to self-referential standards. As users become more attuned to their personal progress metrics rather than external benchmarks, their sensitivity to PE examination stress tends to be lower accordingly.

### 5.3 Factors associated with lower PE examination stress

The decision tree model reveals the factors associatively linked to PE examination stress. Physical self-concept shows the strongest association. Exercise self-efficacy further divides the population into high- and low-efficacy groups. Attitude towards smartwatch use emerges as the final partitioning variable. These findings suggest that positive body image, exercise confidence, and smartwatch attitudes are collectively associated with lower stress, but causal claims cannot be made.

First, the model identifies physical self-concept as the most central associated variable, with the majority of individuals (41%) scoring at a moderately high level (3–4 points), suggesting that a positive body image may serve as the first line of defense against social stress. Second, among individuals with higher physical self-concept, exercise self-efficacy further divides the population into high- and low-efficacy groups, with the high-efficacy group accounting for a significantly larger proportion (27.7%) than the low-efficacy group (13.3%). This indicates that fostering exercise confidence is associated with alleviation of social stress. Finally, within the high-efficacy group, a positive attitude toward smartwatches emerges as the terminal associated factor, providing empirical support for the relevance of wearable technology in promoting healthy behaviors. In other words, on the foundation of improved body acceptance, enhancing exercise self-efficacy and effectively utilizing feedback from smart technology are associated with lower levels of an individual’s perceived PE examination stress.

### 5.4 Individual differences in interpreting digital feedback

The findings of this study must be interpreted within a broader mind-body framework that acknowledges the bidirectional and complex relationship between bodily experience, psychological state, and digital feedback (12, 62). While our aggregate SEM results show that attitudes toward smartwatches are negatively associated with PE examination stress, this average effect may mask substantial individual heterogeneity. For some adolescents, seeing their heart rate stabilize during exercise or observing gradual improvements in step counts may reinforce a sense of interoceptive competence and self-efficacy. For others, however, the same metrics—particularly when displayed alongside peer rankings or normative benchmarks—may intensify body surveillance, shame, upward social comparison, or performance anxiety. This is especially relevant during adolescence, a developmental period characterized by heightened self-consciousness and sensitivity to social evaluation. Therefore, rather than assuming that smartwatch feedback universally benefits all students, researchers and educators should recognize that its effects depend on individual psychological characteristics (e.g., baseline body dissatisfaction, perfectionism, social comparison orientation) and the relational context in which the data are presented.

### 5.5 Educational implications

Based on our SEM results, exercise self-efficacy (β = 0.415) and physical self-concept (β = 0.426) show positive associations with smartwatch attitudes. These findings suggest that smartwatch data might be used to support these psychological factors, though causal claims cannot be made. Educators could consider guiding students in interpreting health metrics such as heart rate and calorie expenditure. Regular data comparisons may help students recognize improvement trajectories. However, specific teaching models such as a “dual-track teaching model” or “digital mirror assessment system” remain speculative and were not tested in this study.

Based on the decision tree analysis, a tiered precision support system could be implemented, tailored to students’ psychological profiles. Support strategies may follow the progressive pathway of “body acceptance → exercise confidence → technology adoption → PE examination stress.” For students with moderate physical self-concept, low-competition activities (e.g., motion-sensing games) may be associated with enhanced body acceptance. Those with high exercise self-efficacy may benefit from peer motivation via smartwatch social features. For tech-savvy students, specialized courses on the “stress-exercise relationship” could be developed. This stratified approach requires periodic reassessment using the decision tree model to ensure support strategies align with students’ evolving psychological needs, ultimately forming personalized stress-management frameworks.

Lastly, traditional PE evaluation systems could be reconsidered by establishing a “digital mirror”-based multidimensional assessment framework. Given the prominent association between physical self-concept and stress mitigation, smartwatch-recorded exercise data could serve as one evaluation metric, replacing single-score rankings with multidimensional radar charts. Virtual avatars generated from students’ fitness data might even act as growth rewards. The metaphor of a “digital mirror” captures how smartwatches reflect objective data back to users, potentially enhancing body awareness and self-regulation. However, a mirror may cut both ways: while it can support self-awareness, it may also intensify self-surveillance and social comparison—particularly during adolescence, when body image and peer evaluation are developmentally sensitive. For some students, seeing their step counts or heart rate data may reinforce a sense of accomplishment; for others, falling short of personal or peer benchmarks may trigger body dissatisfaction or performance anxiety. Therefore, educators should not assume that data feedback automatically benefits all students. Instead, smartwatch integration requires explicit scaffolding—such as disabling peer leaderboards for vulnerable students, emphasizing personal progress over normative comparisons, and fostering digital literacy that helps adolescents interpret metrics as descriptive rather than evaluative. Only with such safeguards can the “digital mirror” serve as a tool for empowerment rather than a source of pressure. This approach not only capitalizes on smartwatches’ data capabilities but also is associated with cultivating positive body self-perception.

Importantly, the effectiveness of smartwatch-based interventions is contingent upon students’ psychological readiness. Factors such as emotional stability, baseline self-esteem, body image satisfaction, and family support significantly influence whether an adolescent can interpret wearable feedback constructively (26). For students lacking this readiness, objective data may become an additional source of pressure rather than empowerment. Therefore, smartwatch deployment in physical education should never be framed as a purely technological solution. Instead, it requires parallel investments in psychological scaffolding—including teacher guidance on healthy data interpretation, parental involvement to reduce performance pressure, and classroom activities that emphasize personal progress over social comparison. Only within such a supportive ecosystem can smartwatches fulfill their potential as a “digital mirror” that enhances rather than threatens adolescent well-being.

These strategies synergize intrinsic psychological capital with exogenous technological support, showing associations with lower levels of stress from peer comparisons and societal expectations while fostering holistic physical and mental well-being. It should be noted that the educational strategies discussed above are speculative extensions based on correlational findings. They have not been empirically tested in this study and should be interpreted as theoretical possibilities rather than validated recommendations.

## 6 Conclusion

The influence of technology on daily life is becoming increasingly pervasive, and using smartwatches to make informed decisions about physical exercise has emerged as a popular choice. Against this backdrop, this study examines the associations between middle school students’ attitudes toward smartwatches and their PE examination stress, providing insights for understanding such stress and promoting the application of smartwatches in school-based physical activity. The results indicate that attitudes toward smartwatch use, exercise self-efficacy, and physical self-concept are all negatively associated with PE examination stress among middle school students. Notably, exercise self-efficacy and physical self-concept show significant negative associations with students’ PE examination stress. These findings offer important theoretical insights and practical directions for understanding and supporting adolescent health.

## 7 Research value

### 7.1 Theoretical values

This study, grounded in social cognitive theory, reveals how attitudes toward smartwatches are indirectly associated with students’ PE examination stress through exercise self-efficacy and physical self-concept, thereby enriching research on psychological-behavioral mechanisms in technology-mediated contexts. While prior studies predominantly examined the impact of interpersonal interactions on self-perception, this work introduces physical self-concept into educational psychology, offering a novel theoretical lens to understand how smart devices are associated with adolescents’ body cognition and exercise behaviors.

Existing research on student stress has largely focused on academic stress, whereas this study specifically investigates PE examination stress and identifies physical self-concept as showing a stronger negative association with such stress than exercise self-efficacy. This finding highlights the unique association between body image and adolescent stress management, providing fresh insights for future correlational research.

The study not only confirms the direct negative association between smartwatch attitudes and PE examination stress but also, through SEM and CHAID decision tree analysis, uncovers the mediating and hierarchical patterns of psychological factors. This model establishes a replicable analytical framework for future studies on how smart technologies are associated with behavior through psychological pathways, particularly offering critical reference value for interdisciplinary research spanning educational technology, health psychology, and sports science.

### 7.2 Practical values

The study demonstrates a negative association between smartwatch attitudes and PE examination stress. This finding offers preliminary empirical support for considering wearable devices as a potential research direction in schools. However, schools should not implement smartwatch-based teaching models without further empirical validation. Future intervention studies are needed to test whether such models are effective.

The decision tree analysis reveals that students with different psychological profiles require personalized interventions. These insights can help PE teachers develop tiered support strategies, such as prioritizing Physical self-concept improvement for high-stress students before gradually fostering exercise self-efficacy and guiding them to use smart technology for autonomous stress management.

Traditional PE evaluations rely on standardized tests, which often exacerbate social comparison stress. This study proposes using smartwatch data to establish a “digital mirror” assessment system that focuses on individual progress rather than peer rankings.

## 8 Limitations and future research

First, this study employs a cross-sectional design, which precludes any causal interpretation of the observed associations. While our theoretical model posits a sequential path from smartwatch attitude to exercise self-efficacy to physical self-concept to PE examination stress, reverse causality remains equally plausible. For example, students who already experience lower stress or possess higher self-efficacy may be more inclined to adopt smartwatches. Future research should employ longitudinal designs with cross-lagged panel analysis to establish temporal precedence, examine bidirectional relationships, and rule out reverse causation (Z. 63). Cross-lagged panel analysis would allow researchers to test whether smartwatch attitudes at an earlier time point predict subsequent changes in exercise self-efficacy, physical self-concept, and PE examination stress, while controlling for autoregressive effects and reciprocal pathways (64). Additionally, the CHAID decision tree analysis is exploratory and descriptive; its hierarchical structure reflects statistical associations rather than causal relationships.

Second, this study focuses primarily on individual psychological mechanisms but does not sufficiently account for external environmental factors. PE examination stress may also be influenced by family support, peer competition, school sports policies, socioeconomic status, parental pressure, teacher support, and baseline physical fitness. The absence of these variables means that unmeasured confounding factors could partially account for the observed associations. Future research should adopt a multilevel analytical framework that incorporates these demographic, psychological, and social-contextual variables to better isolate the unique associations of smartwatch use (65).

Third, this study highlights the positive associations of smartwatch use but does not adequately examine potential negative effects. For some adolescents, continuous self-monitoring may intensify performance anxiety, trigger upward social comparison, or exacerbate body dissatisfaction. The “digital mirror” metaphor implies a usage pattern that we did not directly measure; future research should include process measures such as interviews or think-aloud protocols to verify how adolescents actually interpret smartwatch data. Machine learning and network analysis approaches are needed to identify which adolescents are more susceptible to adverse effects and under what conditions smartwatch feedback becomes supportive rather than pressuring (66). Furthermore, the practical suggestions proposed in the Discussion are speculative extensions of our correlational findings and should be treated as theoretical possibilities for future intervention research rather than ready-to-implement recommendations.

## Data Availability

The original contributions presented in the study are included in the article/supplementary material, further inquiries can be directed to the corresponding author/s.

## Appendix

**TABLE 1:** Measurement items.

Construct	Item
Attitude towards using smartwatch	I believe that my smartwatch will improve my daily life
	I believe that my smartwatch will help me be more productive
	I think I will keep using my smartwatch in the future
	I think smartwatch technology is beneficial for people’s health
PE examination stress	When thinking about conducting PE examination
	I will feel uncomfortable in my stomach
	I always feel uneasy
	I forgot what I had learned because I was too nervous
	I will tremble.
	Feel uncomfortable
	I feel my muscles tense and sore. (Delete)
	Preparing for exams often makes me feel unwell
	I will suffer from insomnia
	I will have stiff shoulders and headaches. (Delete)
	I feel anxious and painful
Exercise self-efficacy	Even though I am very busy with my studies, I can still use my smartwatch to remind myself to exercise every day (such as running and skipping rope).
	I can use the exercise recording function of my smartwatch to find the exercise methods that suit me (such as step counting and heart rate monitoring).
	I can achieve the weekly exercise goals I set with my smartwatch (such as burning 200 calories).
	If I encounter difficulties in exercising (such as bad weather), I can stick to exercising using the indoor exercise mode of my smartwatch (such as aerobics)
	Even if I’m very tired, I will still stand up and move around following the “sedentary reminder” of my smartwatch.
	Even when I’m in a bad mood, I will turn on the “Exercise Challenge” function of my smartwatch to distract myself.
	Even if my friends don’t exercise with me, I can still exercise by myself using the “Personal training mode” of my watch.
	Without the guidance of a teacher, I can still complete the correct exercise through the “action demonstration” of my smartwatch.
	If I slack off for a few days, I can motivate myself to start exercising again with the achievement badge of my watch.
	Even if I don’t go to the gym, I can still do exercise at home with my watch (such as following the APP to do exercises) (Delete)
Physical self-concept	My exercise data (such as steps/heart rate) convince me that I am good at sports
	When checking the daily health report, I appreciate my physical condition
	When my sports data is liked by my friends, I feel very charming
	Classmates often invite me to exercise together through the sports ranking list
	I can effectively adjust my schedule and exercise plan according to the prompts of my watch
	Based on my sleep, exercise and posture data, I consider myself elegant and healthy
